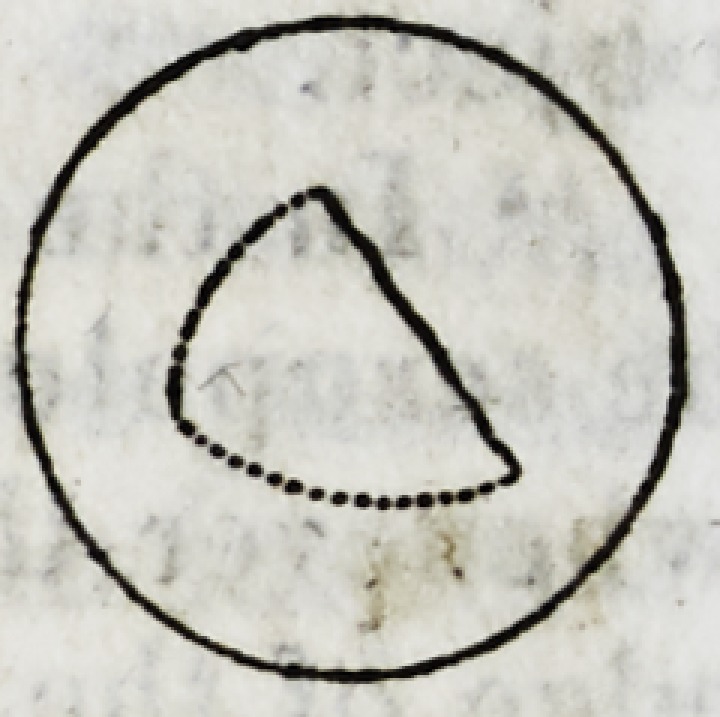# Book Reviews

**Published:** 1817-06

**Authors:** 


### CRITICAL ANALYSIS

### *OF RECENT PUBLICATIONS,*

IN THE

### DIFFERENT BRANCHES OF PHYSIC, SURGERY, AND MEDICAL PHILOSOPHY.

*A Statement of Circumstances connected with the Apothecaries' Act, and its Administration.* By GEORGE MAN BURROWS, M.D. F.L.S. &c. 8vo.—Pp. 69. Callow, London.

THE progress of the sessions, the importance of the subject, and the manner in which the author of this statement has contrived to give it publicity to one class of readers, induce us to offer the following abstract of the question in addition to our customary sheets. It is not necessary that we should follow the author in an egotism of which, at last, he becomes sensible himself. It will be enough, if we state in as few words as possible the supposed imperfections of the Act, the consequent difficulties in fulfilling some of its intentions, and the *desiderata* still remaining before those important purposes can be accomplished.

The first eight pages are taken up with an historical account of the progress of the Association for rendering the practice

"* Active measures are now taking to remove this cause of complaint."

practice of the apothecary more respectable, the difficulties which presented themselves, and, lastly, the determination of the legislature to invest the intire control in the hands of the Society of Apothecaries, whose court of assistants have the power of appointing a board of examiners.

"The period (says Dr. B.) had now arrived when the members of the Court of Examiners were to be nominated. I received a summons to be sworn in as one of them. Report informed me of the names of some others; but I knew not who they all were until they assembled to take the oath of office before the Court of Assistants. They were all gentlemen who, I was confident, were fully fitted for the office; but there were two members of the Court of Assistants among them! The inauguration was altogether ominous. Some of the gentlemen who were summoned, were so little interested in the matter, that they did not know such an act was in existence, till they were desired to take upon them the office of examiner. They objected, that, having never seen the act, which, by the bye, ought certainly to have been sent with the summons, they did not know what they were called upon to undertake or be sworn to do. However, presently all were summoned before the court, and were ranged standing in a rank. The clerk read the oath, each kissed the book, and then walked out just as wise as he entered. Not a syllable was spoken, no explanation of the nature of the functions we were called upon to exercise, no civility, no compliment; in short, the most important occurrence that ever happened within those walls, passed as a trivial and ordinary affair!

"When withdrawn to another room to elect a chairman, &c. Mr. Simons, a member of the Court of Assistants, as well as of the new court, placed himself in the chair, pretty plainly indicating that that was his destined place. He was accordingly immediately and unanimously elected,—a compliment, I admit, which certainly was due to him, and could not have been dispensed with. He informed the court, that the Court of Assistants had been pleased to appoint Mr. Watson, his son-in-law, secretary to the Court of Examiners! To the gentleman nominated as secretary none could object; for one better fitted for the office, in every particular, could not have been selected; and I beg to observe, that his future conduct merited every praise."

We are next informed, that, finding no bye-laws prepared for regulating their own meetings, Dr. Burrows proposed, and succeeded, though not without some opposition, in procuring a committee of himself and two others, for drawing up a code.

The remuneration for the *examiners*, and for the company's clerk and beadle, was next fixed by the Court of *Assistants.* All this appeared derogatory to some of the examiners, especially as the notice was only given verbally by their chairman, who was also a member of the Court of

 Assistants,

Assistants. Dr. B. considering a verbal communication from a court which was to direct their proceedings as irregular and insufficient, procured a resolution, requesting that all future communications should be in writing.

It is not to be wondered, if on a subject so entirely new and untried, an act obtained under so many difficulties and disadvantages, should prove inadequate in some points. The first of these was a severe clause introduced, we are informed unintentionally, that no person should be admitted to examination who had not served an apprenticeship of five years. This, and some other imperfections, induced Dr. B. to make two motions to the following effect:

1st. That it has become manifest to this court, that, contrary to the intentions of the society, and prejudicial to the public, there are numerous errors and defects in the Act for the Regulation of the Practice of Apothecaries.

That a Memorial shall be presented to the Court of Assistants by this court, expressive of the preceding resolution; and that a committee of such persons as are most conversant with the provisions of the act, is requisite to revise the same.

The first only of these resolutions was past by the Court of Examiners.

A still greater difficulty followed, "Counsel had given an opinion, that no army or navy surgeon could practise as an apothecary in any part of England or Wales, unless he had been in practice as such prior to the 1st of August, 1815." As the court felt no disposition to attend to this and some other difficulties, Dr. B. thought it his duty to address the Court of Assistants, and, presuming they might be uninformed of the difficulties the examiners experienced, the following papers were sent to them.

"OBSERVATIONS ON THE APOTHECARIES' ACT.

"SECTION 3.—Excepting as far as regards the examination and destruction of bad drugs, this section is nugatory; because, although a penalty of £5 is imposed for the first offence, yet it cannot be recovered from the error in drawing up Section 26.

"SECTION 9.—The obligation for the examiners to meet once in every week is objectionable, and may often be attended with great inconveniencies. At any rate, there ought to be a discretionary power or latitude given, that the court should not be obliged to assemble, whether there is business or none.

"SECTION 12.—There should be power vested in the Court of Assistants to administer an oath to every secretary to the Court of Examiners; that office being one of great trust and confidence.

"SECTION 15.—The omission of a single sentence in this section, renders the act retrospective, and therefore unjust; in the clause in the original bill, after the words 'that no person,' there followed lowed in a parenthesis ('except such as shall be actually bound by proper indentures or shall have commenced a course of medical education, at the time of passing this Act.') The restoration of this sentence, and referring the operation of it antecedent to August the 1st, 1815, would rectify this unfortunate and grievous oversight.

"As few youths, apprenticed to country apothecaries, have opportunities of acquiring a proper knowledge of the fundamental principles of their profession, the imposing of a term of five years is manifestly injudicious, and tends rather to prevent than facilitate their proficiency.

"SECTION 16.—To comply with the provisions of this section, an evasion of the letter of the Act must be practised, or the master, wardens, and Society must meet weekly; for otherwise, *their* clerk cannot signify *to them*, the notices of persons intending to qualify.

"As this is the only duty imposed by the Act on the clerk of the society, and may be wholly dispensed with, a trivial alteration of words would obviate the difficulty.

"SECTION 17.—This section is altogether nugatory, from the error in section 26, as is proved by the experience of six months since the passing of the Act.

"SECTION 18.—This also is nugatory until section 26 is corrected; nor could it be carried into effect if that section were perfect; because,

"1st, Monthly meetings of country examiners of assistants are enjoined, whether there is business to transact or none.

"2nd, No fund possessed or acquired under the Act, would be adequate to remunerate respectable practitioners of ten years standing, for acting as examiners.

"3rd, As there is no provision to induce them to act voluntarily, and nothing can compel them, such offices will never be executed.

"There is no mention in the Act of any person to whom country assistants are to apply, when desirous of examination. The 16th section refers to those, only intending to practise as *apothecaries*.

"SECTION 20.—The greatest presumed offence of which the Act takes cognizance, is, practising without examination and a certificate of competency; and yet the fine imposed is £20 only on persons so offending, while by section 5, the non-compounding of a physician's prescription, or unfaithfully compounding it, makes such offender liable to information before a magistrate, punishable by pecuniary fines equally large, and, moreover 'be rendered incapable in future of using or exercising the art and mystery of an apothecary!" Now this is inflicting for the minor a punishment infinitely heavier than for the major offence; contrary to sense and justice, and the custom of the English law. The degree of penalty in this section (20) should be regulated by the enormity of the the offence committed. Either the penalties here are too trivial, or, in section 5, they are excessive.

"This section also imposes a penalty of £5 on every assistant acting without a certificate. But no penalty attaches to persons for employing them without this proof of their having conformed to the Act.

"SECTION 22.—No person is mentioned to whom a candidate should apply, who is desirous of re-examination.

"SECTION 26.—From the omission of two words this section, as far as regards the recovery of penalties or forfeitures of the sum of £5 or under, which particularly applies to assistants, is nugatory. The ninth line should have the words, in the parenthesis, added, 'and if such penalty or forfeit shall amount to *(or be)* less than,' &c.

"This defect actually makes void all penalties on assistants; and is probably one reason none have yet applied for examination for a certificate.

"SECTION 28.—It is generally conceived that this section does not prevent those who were druggists and chemists, before the passing of the Act, from prescribing and practising medicine. As that was most assuredly the intention of the legislature, all obscurity of the meaning of this section should be removed.

"If any information or action were brought by the society against any offender for practising as an apothecary without a certificate, it has been suggested by good authority that a question might arise of the meaning of practising '*as an apothecary*,' and what the real functions are of a person so denominated.

"Does practising, by visiting and prescribing for the sick without a licence from the College of Physicians, or the mere keeping of a shop for the dispensing of physician's prescriptions and the compounding of medicines, constitute a lawful apothecary?

"If this question cannot be answered positively, an amended Act should guard against such a question being ever raised, by reciting specifically, and defining what is the practice of an apothecary.

"There are several other errors, of lesser importance, perhaps, which cannot fail of presenting themselves to a committee of persons well conversant with the provisions and omissions of the act.

"*March* 26*th*, 1816. (Signed) G. M. BURROWS."

A letter follows containing many additional arguments for an application to Parliament to amend the Bill in all the above particulars, and in some others of less importance. No notice was taken of these papers by the Court of Assistants; but, in consequence of a memorial from the army and navy surgeons, Lord Palmerstone was preparing an Act to relieve those gentlemen from the necessity of examination by the Society of Apothecaries.

We come now to what, in our opinion, is the most important part of the whole.

"In

"In the course (says our author) of the first ten months of the labours of the Court of Examiners, they had seen, with great regret, the deficiencies of the candidates who offered for examination; that many had consequently been rejected; and that others, who had passed, were by no means so perfect as could be desired, especially in chemistry, pharmacy, and materia medica. Nothing is more imperative than that every one who prescribes remedies for diseases should be acquainted with them when unsophisticated and in their natural state, as well as with their qualities, doses, &c. Candidates were perpetually pleading, as an excuse for ignorance, that, in the shops where they had served, all chemical preparations, tinctures, compounds, &c. were purchased ready prepared; and that drugs, such as bark, rhubarb, &c. were always in the form of powder. Many other important defects evidently existed in the elementary part of their medical education. The Court, sensible of this, drew up and published some very judicious regulations as to the nature of, and the testimonies of education which they expected from students before they presented themselves for examination. But, aware of the physical impediments which prevented young men from acquiring, while apprenticed in the country, proficiency in this elementary knowledge; and that when they came to London they thought these studies of such inferior consequence, that they seldom paid them any attention; I entertained a belief, that, if a degree of emulation could be excited among the students, the study of medicine, chemistry, pharmacy, and materia medica, might be equally and as ardently cultivated as anatomy and surgery,—to which, almost exclusively, the majority devoted themselves.

"The desultory mode of education pursued in the London medical school is, for many reasons, objectionable; but chiefly because talents are never fairly brought into collision; consequently, emulation is never excited. Hence, too, extraordinary merit meets with little notice beyond the insulated limits of a single hospital, and is long before it receives its just reward from public patronage.

"I had before delivered my opinions very fully on this subject, in the review of Cross's Sketches of the Medical Schools of Paris, in the Medical Repository.

"I knew it was impossible for the Court of Examiners, which was not the executive body of the Society of Apothecaries, to carry any extended plan into practice; but I felt that, as a constituted body, it could, at a trifling pecuniary sacrifice, set an example that would confer everlasting honour upon its public character, and a most essential service both upon the public and the profession. I therefore had arranged a few outlines, which, as the first year of the services of the Court of Examiners was near expiring, and the same members might not be re-appointed, it was a fit time to propose. On June 13th, therefore, I submitted an introductory motion—

"Prefaced by a few general remarks, stating, that, as the examination examination embraced physiology, the practice of medicine, chemistry, pharmacy, materia medica, and was also to include botany, that, if a prize were offered annually for an essay on one of these subjects in rotation, that each would be treated once in every five or six years; and thus time would be allowed between every essay for new facts and discoveries being developed and collected, which would always afford fresh matter to the prize subject, whenever it revolved. And, if it were thought this proposal would be objected to by the Court of Assistants, as there were twelve Examiners, a subscription of a few guineas each would furnish a prize of sufficient value to excite attention and competition; and establish a precedent worthy the example of their successors."

Without inquiring what is meant by *physical impediments*, the above passage is enough to show how much more easy it is to condemn than to amend. That the candidates should be found deficient in many points, can never be a matter of wonder when we consider what is expected of men at the age of twenty-one years. Let us ask which of those who have passed the most brilliant ordeal at either of the Royal Colleges in London, would be equal to examinations, in a single day, on physiology, the practice of medicine, chemistry, pharmacy, materia medica, including botany. If it should be urged that these examinations are not severe, some would suspect them to be useless. It should be remembered that the Court contains its own professor of botany, its members of the Committee of their own elaboratory, several members of the College of Surgeons, and gentlemen of such experience in a practical art, as must render their opinions authoritative to the youths submitted to their examination:—How fit soever such men may be to form a Court of Examiners, may not this very fitness increase the difficulties of the examined?

To remedy these inconveniences, Dr. B. proposes schools of emulation and prizes, in imitation of the French. These are most unfortunate suggestions. Whatever superiority that nation may claim in the manual part of practical surgery, do they not crowd to the British schools for instruction in medicine? Do we not see enough of schools of emulation in the Medical Societies of the London and Edinburgh students, where the principal object seems too often the embarrassment of the most modest, though not always the least informed. Still less are we disposed to encourage the distribution of prizes. Men who are expected to be well-informed on so many points, could only incur a certain loss of time by devoting it to the only subjects for which prizes could be instituted. They would not, surely, propose the practice of physic as a prize to youths of this age; and, in becoming complete botanists, chemists, or even even physiologists, is there not great danger of their undervaluing pathology, the sole purpose of all their studies, if intended to render them skilful practitioners? We are, therefore, much less surprised than the author that these propositions were negatived, and even that they had not the support of half the Court.

From this time we learn nothing but of the discordant sentiments of the author and his colleagues, till we arrive at the event noticed some time past by a letter in our Journal for January last.* The reader will recollect, that, on the insertion of this letter, we never gave it as genuine. We could not conceive it possible that such a document could, by any accident, come before the public. Yet, as the case was one which might happen, and even produce a difference of opinion on the construction of the act, we thought it our duty not to withhold this or any other information on so important a question. We shall now transcribe all that we can collect from this tract concerning the manner in which that paper came before the public.—The author, finding no notice taken of his Letter by the Court of Assistants, addressed the Master personally. That gentleman's situation was somewhat delicate, as he was also Chairman of the Committee, and bound by his oath, as Master of the Company, not to reveal the secrets of the Court.

"I now felt assured (says Dr. B.) that no notice would be taken of my letter of Oct. 3d. Having waited till December, I was almost resolved to lay the whole transaction before the public, but from this I was dissuaded; it being urged that it was very possible that the Court of Assistants had been so much occupied with other business that they might not have had time to enter upon the subject; and that, as there would be another Court at the latter end of that month, it would be prudent to wait the event of it. To this advice I gave assent. When, on the 1st of January, I found I was completely anticipated; for my letter appeared in the Medical and Physical Journal, and in the Medico-Chirurgical Journal. The Editors added, that it was anonymously sent; and yet, in each Journal it is differently inserted. I suspended my intention of publishing to see what would be the result. A considerable sensation was evinced by those who felt themselves exposed to censure by the publication. The Court of Examiners took no notice of it; but, nevertheless, great inquiries were made to discover the publisher. I found that I was suspected; but that little concerned me. I frankly answered all my private friends who spoke to me on the subject, and contemned the idea of its being imputed particularly to me, when there were so many other channels through which it might have become public."

* See page 78 of this vol.

We

We confess we should have been better pleased with a positive answer from the author, that it was published without his knowledge or consent, and that he still remained ignorant of the manner in which it had found its way into our Journal. For ourselves, we assert, on the responsibility of our Editor, that we are ignorant of the channel by which it reached us, or why it was withheld from his (Dr. Burrows's) own Journal, and published differently in two others. It cannot be wondered if Dr. B. was afterwards summoned to appear before the Court, in order to account for the manner in which the letter had been made public. An account of the inquiry before the Court follows, which can only amuse those who delight in seeing the awkward situation in which the best-intentioned men are sometimes placed; for who could expect the publication of such a document, and who could fail, when it appeared, to suspect the source from which it might come? To conclude this part of our subject, we cannot help remarking, that the *ex-parte* publication of this conversation and of the first meeting of the Court of Examiners, argues less attention to delicacy than even the publication of the document in dispute. The conversations are authenticated in part: the document might have passed for a mere contingent case, pointing out one of the imperfections in the Bill.

Such is the history of this dispute on a subject of such high importance to the health of almost every British subject. That the Bill is imperfect, cannot be questioned; but that it might have been amended by a disposition to accommodation in those to whom its execution was committed, we can hardly doubt. What the issue of it may now be, we know not; but we recommend to all parties a sentence we remember to have read in the Hall at Blackfriars. As it is blazoned on glass, we presume it was preserved from the ruins of the great fire; and we trust no one will dispute how worthy it is of the care with which it has been restored.

"Concordiâ parvæ res crescunt; discordiâ magna dilabunter."

---

*Medico-Chirurgical Transactions, published by the Medical and Chirurgical Society of London.* Vol. VII. Part II.—Longman and Co. 1816.

*(Continued from p. 337.)*

*Observations and Cases relating to the Operation for Artificial Pupil; in a Letter from Mr. Maunoir of Geneva, to Professor Scarpa of Pavia, with the Professor's Answer.* Communicated by Dr. MARCET.

OUR Journal has already been the means of offering to the world M. Maunoir's opinions concerning an operation on this this most delicate part of the delicate organ of vision.* The present paper contains two cases, we doubt not faithfully related; but, as in the long course of our practice, we have never engaged in such an operation, we shall only complete our former paper by extracting Scarpa's general summary of the improvements of others, and those suggested by himself.

"Every lover of the science (says the learned professor,) cannot but feel interested in the narration of the difficulty which attended this operation, as well as that performed on Mrs. Saillard, on account of the opake crystalline, which in both cases adhered to the lower circumference of the iris, and the edge of the closed pupil. This has given me occasion to make some reflections on the subject, which I take the liberty of communicating to you with my usual freedom.

"The detail of both the operations in the above-mentioned cases, exposes a certain degree of difficulty and uncertainty in the proceeding, which I should wish to see removed or corrected; and I have great hopes that you will be able to attain this desirable object.

"In the first place, I am of opinion that it is not necessary to be scrupulous whether the crystalline be partly or entirely opake, whenever the capsule is opake, and adheres to the iris behind the edge of the interior and inclosed pupil. In this case, only one remedy can be pointed out, namely, the removal of the opake adherent capsule, and consequently of the crystalline, whether it be transparent or opake.—In the second place, I think there is no reason to doubt that, in similar cases, it is advisable to make an incision upon the iris proportioned to the size of the body to be extracted, rather than to make it small, which obliges the operator to divide the crystalline and the capsule, with the intention of extracting a part, and of abandoning the rest to the powers of absorption.—Thirdly, I would establish as a fundamental principle in similar cases, that after the complete extraction of the crystalline with its opake capsule, by means of the least possible introduction of instruments, the artificial pupil ought not to be too near the incision in the cornea, and, consequently, not too near the cicatrix occasioned by it.

"The causes of the obstacles, to which you were exposed in the two cases above-mentioned, may, I think, be perceived from a consideration of the principles I have just stated. In both, the incision in the iris was too small in proportion to the size of the body to be extracted; and in both, the position of the artificial pupil was very

* See in our XVIIth Vol. p. 403, two Memoirs on the Organization of the Iris, and on Artificial Pupils; by Mr. T. P. Maunoir, Surgeon of Geneva. Translated by Mr. Thomas Young, of London, with engravings.

disadvantageous; that is to say, on the side of the temple and close to the incision in the cornea.

"After reflecting attentively upon this situation of the artificial pupil, and upon the obstacles which it presents to the operator, it appears to me, if I am not greatly deceived, that a method of operation compounded of that of Wenzel, and of your own, would perfectly answer the desired end. Wenzel, as you are aware, made an incision upon the cornea and the iris with a single stroke of his instrument, taking care that this transverse incision should pass through or underneath the centre of the inclosed pupil. He then took off, by means of the scissors, a portion of the edge of the iris, for the double purpose of extracting with facility the opake crystalline with its capsule, and of leaving a permanent artificial pupil of sufficient size. In the method which I would suggest, after having made, in the manner of Wenzel, a transverse incision in the iris and in the cornea, I would introduce your scissors, blunted at both points, into the anterior chamber of the aqueous humor, with which I would make an incision in the iris, diverging from the cut made by the knife, so that your usual triangular edge might be the result, having a curvilinear side. This aperture, which requires only a single stroke of the scissors, will be, I think, sufficiently large to allow easy egress to the crystalline and the capsule; and this so much the more easily, in proportion as the point of adherence of the capsule to the iris, is comprehended either entirely, or in a great measure, within the two incisions. By this means the facility of making the crystalline and the capsule pass obliquely out from the iris will be increased, on account of the enlarged space that will result from the cut with the scissors diverging from that made by the knife; and I should prefer this incision with the knife to the puncture made by you in the iris of Saillard, to afford a passage to the blade of the scissors. Besides this, the direction and the situation of the triangular edge of the iris will be calculated to leave a pupil not only permanent, and sufficiently large, but also placed opposite to the cut in the cornea, and accordingly more convenient for the purposes of vision; especially if it fall upon the side of the iris nearest the nose, which ought, if possible, always to be the case.

"I have thus briefly given you my opinion on this subject. You will recollect that you permitted me to make objections and suggestions. I have done so; and it now belongs to you in the course of your practice to make trial of the method I propose, and either to confirm or reject it, or, which will not be difficult to you, to suggest some new and more practicable expedient. It is certain that this complicated case of the pupil, that is to say, where the crystalline is found opake, together with the capsule adhering to the iris, requires an exertion of genius and skill, united to a more perfect method of operation, than that which has been hitherto practised. Your researches have already been so numerous and so successful in this branch of our profession, that we have a right to to expect you to proceed in the completion of your work. Till within a very few years, our knowledge respecting the artificial pupil, and the method of conducting the operation, was involved in great obscurity, and practice was sometimes even in opposition to the known anatomy of the eye. It is by solid and fixed principles alone that we ought to be guided in all that variety of complicated cases, which frequently accompany and aggravate the inclosed pupil. In estimating the extent of the services which you have already rendered to the profession, I run no risk of error in enumerating the following facts as the results of your most useful researches.

"1. That no instrument is so proper as the scissors for making an incision in the iris.

"2. That to do this, when there is no complication resulting from a cataract, a very small incision in the cornea is sufficient, about half the size of that which is made for the extraction of the crystalline,—a fact which on many accounts is of the highest importance.

"3. That the formation of a triangular edge in the iris by means of a double incision with the scissors, is the most easy and least painful of all the methods hitherto proposed for obtaining a permanent artificial pupil.

"4. That the spots of the cornea present no obstacle, because it is possible to produce the artificial pupil in that part of the cornea remaining transparent, in the quarter opposite to that in which the incision is made,—a fact of the greatest importance.

"5. That it is possible to obtain the artificial pupil without injury to the crystalline or its capsule, whenever these parts are preserved transparent, in spite of complete confusion in the iris.

"If to these advantages, which your method of operation possesses over all those hitherto practised, you are able to add that of rendering as little laborious as possible the manner of making the artificial pupil, in those cases where it is necessary to remove at the same time the crystalline and its opake capsule adherent to the lower surface of the iris, you will fulfil not only all my wishes, but those of all who are interested in the cause of humanity, and in the progress of science. The state of my health does not permit me to follow you in this useful career, and leaves me only the power of witnessing and of applauding your success. I have much pleasure in informing you that your scissors have begun to be used here, and Signor Morigi, my successor in the chair of surgery, has lately availed himself of them with great success in an operation for the artificial pupil. He is a man of considerable ability, whom you will find mentioned with deserved praise in several places of my work.

"I have the honor to be your most sincere friend and humble servant, "A. Scarpa."

As it is well known, that Professor Scarpa has been for many years the consulting surgeon of all Italy, at one time the first anatomical school in the world; as we had practical proofs of his candor and diligence during his residence in

London between thirty and forty years past; and, as in the whole of his letter, he offers no *practical* remarks from his own experience, we are the less scrupulous in acknowledging, as before, our entire ignorance of the success of the operation here proposed.

*Case of a Wound of the Peroneal Artery, successfully treated by Ligature.* By George James Guthrie, Esq.

Not only the situation of the artery, but the quantity of extravasated blood, and the slough always induced by the passage of the ball, rendered this operation extremely complicated; and its happy issue does credit to the author of the paper.

*Case of a Gun-shot Wound and Fracture of the Tibia, in which a Seton was successfully employed in promoting a Cure.* By John Boggie, Esq. Surgeon to the Forces.

"Insulated cases often become extremely valuable in establishing general doctrines, or in illustrating practical rules in medicine; for, by supplying one link in the chain, they enable a series of facts to be classed together, with which no connexion had previously been established. On this account, the following case may not be considered unworthy of being read before the Society; for, although the use of a seton in this instance could have afforded no probable grounds for suggesting the practice as a general one in ununited fractures, yet it shews the utility of it under particular circumstances, and may illustrate some of the facts and observations which have been made on the subject by Mr. Wardrop, in the fifth volume of the Transactions of this Society."

*An Account of a new Method of Operating for the Cure of external Aneurism, with some Observations and Experiments illustrative of the Effects of the different Methods of procuring the obliteration of Arteries.* By Philip Crampton, Esq. F. R. S. Surgeon-General to the Army and Forces in Ireland.

This is a most important paper, written with much candour, and involving so many questions, that we shall make no apology for the space we may occupy in our remarks. The author begins, according to the custom of most writers, with hinting (to do him justice very briefly) at Petit, Pouteau, and Kirkland. He then carries us on to Dr. Jones, whose labours have occupied so much of our Journal.*

* See vol. xv. page 463, and vol. xvi. page 87 (in the latter, at line 24, for *twice* read *of twine*). If the reader wishes to understand either Mr. Crampton or us, we must intreat his refreshing his memory with a perusal of the above article, unless he is master of Dr. Jones's opinions.

After a candid examination of that gentleman's book, he concludes, "that analogy between brutes and man is uncertain, and that

"1. It may be stated in general, that the adhesive process is more quickly and certainly executed in all the parts of quadrupeds (with the exception of the skin) than in man.

"2. In quadrupeds, wounds of the arteries in particular are so prone to unite, that no experimentalist has hitherto succeeded in producing an aneurism in this class of animals; the wounds of the arteries which have been inflicted with this view, healing like wounds made in any other part of the body.

"3. The arteries of quadrupeds are not liable to that peculiar change of structure from disease which predisposes to aneurism, and which, among other causes, renders the operation of the ligature so uncertain in its effect upon the arteries of man."

Aneurism Mr. Crampton considers a disease peculiar to man; and that, however successful the operation proposed by Dr. Jones may be in brutes, it is far from being invariably so in man. This is imputed to a difference in the structure of arteries in the two orders of beings. We are not aware that this difference has been ascertained by any accurate examination of the structure of each. It is also certain, that many experiments on the intestines have been made successfully in dogs, which have proved fatal in men; yet no one conceives any difference between the structure of the two. However, it is always proper to keep in view an observation of Mr. Hunter, that the usual pulse of a horse is about half as frequent as that of a man;* so that, the force by which the artery is dilated not returning so quickly, the recovery of the arterial figure may not be compleated by the next pulsation. At the same time we should reflect, that many other experiments may be made in brutes which it is not safe to attempt in men.

Mr. C. next proceeds to shew, from a variety of observations and experiments on the arteries of man, as well as of other animals,—

"1. That the obliteration of an artery can very certainly be effected, independently of the rupture or division of any of its coats.

"2. That this operation of the ligature, so far from being essential to the process, not unfrequently defeats it.

"Numerous instances are on record of arteries being obliterated by the pressure of tumours. The subclavian and carotid have

* Treatise on the Blood, p. 151.

been been found obliterated by the pressure of an aneurism of the arch of the aorta.*

"In Mr. Freer's experiments, the pressure of a tourniquet for four days was sufficient to effect the obliteration of the radial artery in horses.†

"Mr. Hunter observed, that in dogs the mere exposure of the tibial artery to the air for about an hour, excited such a degree of inflammation and thickening of its coats, as completely to obstruct the canal.‡

"All the great arteries, the aorta inclusive, have been found obliterated in consequence of the effusion of lymph from their internal coat, and this independant of any injury which could produce the rupture of that membrane.

"The cure of aneurism by compression (whether mediate or immediate) affords an example of the obliteration of an artery, independant of any rupture of its internal coats.

"The following experiments, selected from a great number which were performed upon the arteries of horses and sheep, will be sufficient to prove, that, in quadrupeds, the obliteration of an artery can be as certainly effected without any perceptible injury being inflicted on its internal coat, as when that membrane is completely divided by the ligature."

Two experiments are next described, shewing that mere pressure is, in the sheep, sufficient to induce the obliteration. Before we consider these, and the inferences of the author, we must detain our readers on two subjects. The first is the quotation from Mr. Hunter. We wish the reader to remark, that, contrary to the other notes, no reference is made to the exact passage. This we shall supply, giving, at the same time, the exact words of Mr. Hunter. "The posterior tibial artery of a dog being laid bare, and its size attended to, it was observed to be so much contracted in a short time as almost to prevent the blood from passing through it; and, when divided, the blood only oozed out from the orifice."§ If this is not the passage alluded to, Mr. Crampton is answerable for our error in not directing us better. If it is, we beg to ask by what authority Mr. Hunter is made to say, "the obstruction to the canal was the effect of inflammation excited to a high degree." We meet with no such expression or inference in Mr. Hunter. On the contrary, his whole object is to prove the muscular power of the artery,

"* Hodgson, p. 110.—Medico-Chirurgical Transactions, vol. i. p. 12.

"† Obervations on Aneurism, p. 14.

"‡ Hunter on the Blood, &c."

§ Treatise, p. 114.

and and the circumstances under which the power is sometimes exerted beyond the degree necessary for the ordinary actions of the part. For this purpose, he evidently shews that no inflammation is necessary; for the next paragraph informs us, "That, in laying bare the carotid and crural arteries, and observing what took place in them while the animal was allowed to bleed to death, these arteries very evidently became smaller and smaller." The fact was still more strikingly proved in the placental arteries, which, even out of the body, and when cut transversely, contracted so as entirely to close the area. All this, Mr. Hunter shews, is the effect of a living process, and, like many others, acts according to the necessities of the animal.

As an artery derives its vessels of nourishment from the surrounding parts, there is always danger of its life when denuded: it therefore contracts, and the danger of death from hæmorrhage, should the portion of artery slough off, is thus prevented. When an artery is divided, it attempts to contract its diameter; and this attempt, in all but the larger arteries, is often sufficient to stop the hæmorrhage. The placenta, from the nature of its functions, and from the danger to which the animal would be exposed were its vessels to continue open after the separation of the child, is probably endowed with arteries possessing the greatest power of contraction. In Mr. Hunter's experiment, he found contraction commence immediately after the chord was cut. This he imputed to the elastic power, but the day following he found the mouths entirely closed. That this last action could only be effected by muscular contraction, he afterwards proved, by shewing, that, if the operation was performed after a certain time, when the parts have lost their life, and, of course, the muscles cease to contract, their mouths would remain open.

Mr. C. next gives it as his opinion, "That the rupture of the internal and middle coat of an artery not unfrequently prevents the obliteration of the artery, and thus gives rise to secondary hæmorrhage. This opinion rests on an assumption that rupture of the internal coat is the *immediate* cause of aneurism, and that the predisposing cause is to be found in certain morbid changes peculiar to man, admitting that the ligature produces such a rupture. Why then, continues the author, does not aneurism more frequently succeed to the rupture of the middle and inner coats? The answer is, sometimes it is prevented by adhesions of the ruptured surfaces, sometimes by sloughing, and sometimes a dilatation of the artery follows, which he considers as similar to an aneurism. In answer to this, we would urge, that; that, though secondary hæmorrhages do sometimes occur, yet they are, comparatively speaking, uncommon, and have been more so since the custom of using round and sharp ligatures has been more general. That, if they still sometimes happen, it is by no means with the frequency that should be expected if the rupture of the internal and middle coat of the artery were sufficient to induce them, and if sharp round ligaments were sufficient to induce such ruptures. However, the conclusions drawn from the above premises are—

"1. That we are not warranted in concluding 'that the internal and middle coats must be cut quite through all round the artery, in order to procure the adhesion of its sides,'* but merely that adhesion may take place under such circumstances.

"2. That, *in man*, the rupture of the internal and middle coats by the ligature not unfrequently gives rise to aneurisms, or perhaps to secondary hæmorrhage.

"3. That a very moderate degree of irritation applied to the *external* coat of an artery, aided by a sufficient degree of compression to bring its internal surfaces into contact, is sufficient to effect the obliteration of the canal.

"4. That the permanent obstruction of the canal may be effected by such a process in a period not exceeding twenty-four hours."

In proceeding to make the application of these aphorisms, the author begins with remarking the danger of the operation, and, on the authority of Mr. J. Bell, asserts that "Hunter himself has lost his patients." We do not recollect the history of these unsuccessful cases in an operation which the inventor had not introduced many years before his death. "Mr. John Bell's *spirited* description of the danger attending the Hunterian operation" is afterwards mentioned as "somewhat overcharged." It would, then, we conceive, have been much better to have omitted such authority altogether, especially as no arguments were necessary to shew, that, however safe the operation may be, it is still the duty of every surgeon to compress the artery, not only, if possible, "without inflicting any injury on the diseased vessel," but even without any use of the knife whatever.

Some remarks follow on the spontaneous cure of aneurism. As these are only conjectural, and sometimes obscure, we shall not detain the reader with them, but proceed at once to the relation of those cases by which "it would appear that the temporary compression of an artery, upon what-

"* Jones, p. 170."

ever ever principle it may act, may be successfully employed for the cure of an aneurism."

In both these cases, the artery was laid bare, and compressed by a ligature so connected with an instrument as to be readily lightened or loosened. The circulation was impeded from 19 to 24 hours, after which the ligature was removed, and the artery became obliterated.

Now, we can refer our author to a paper in our Journal, in which he will find the practice of pressure much simplified, yet equally successful. By means of an elastic steel, pressing on a grooved piece of wood similar to that used with the tourniquet, and applied on the sound skin over the artery, the blood coagulated in a diffused aneurism. This, however, did not appear sufficient to cure the complaint, till, by a fresh application of the same instrument, continued for a few hours, the artery was completely obliterated.*

We shall conclude by transcribing the author's postscript, with a few remarks of our own.

"Since the preceding observations were written, a case of the greatest interest has occurred in one of the hospitals of this city, and which I am permitted to communicate to the Society.

"'On Friday the 23d of February, 1816, the femoral artery was tied (in the manner recommended by Mr. Travers) for an aneurism of the posterior tibial artery. On Monday the 26th, the case appeared to be doing well, all pulsation having ceased in the tumour; and the temperature of the limb being natural, a slight attempt was made to loose the knot, which, proving ineffectual, was not persevered in. On Wednesday the 28th, at eight o'clock A.M. the ligature, appearing to lie loosely in the wound, was withdrawn without resistance, the loop remaining perfect: a violent hæmorrhage came on at one P. M. the same day.

"'The artery was secured again about three inches higher up. No alteration in the temperature of the limb followed this operation. On Friday night the patient became delirious, and died on the following morning.'

"On dissection, the artery was found to be completely divided at the place of its first ligature: its extremities had retracted to the extent of three quarters of an inch. The mouth of the upper extremity of the tube was circular, its area was not contracted, nor were its coats thickened; but it was imperfectly obstructed by a coagulum about half an inch long, which seemed to adhere partially to the internal coats. There was no appearance of lymph having been effused from the cut edge of the divided coats. The

* See Med. and Phys. Journal, vol. vi. p. 535. "The tumour was so perfectly solid, that there was no doubt the blood had coagulated." P. 537.

lower extremity of the tube presented similar appearances, with this difference, that it was more perfectly obstructed by a coagulum.

"It would seem, then,

"1. That the division of the internal and middle coats had been effected in the usual way by the application of the ligature.

"2. That the complete division of the tube, in consequence of the sloughing or ulceration of the external coat, had taken place *at least* on the morning of the fifth day; for, on that day, the ligature with its loop lay loosely in the wound, and was withdrawn without resistance.

"3. That, *after the division of the tube*, hæmorrhage was delayed by the presence of a slightly adherent coagulum.

"The striking feature in this case is the occurrence of hæmorrhage on the fifth day: how far this accident is to be attributed to the attempts, however cautious, to withdraw the ligature from an artery already weakened by the division of its internal coats, I do not pretend to determine; but that a vessel so circumstanced is not in a condition to bear violence or even disturbance of any kind, will, I believe, be readily conceded."

We come now to sum up our remarks with as much brevity as is consistent with perspicuity. From Dr. Jones's experiments, it is unquestionable, that, after the application of a ligature so tight as to divide the internal and middle coat of an artery, effusion of lymph followed, and the obliteration of the calibre. It is true, this experiment was only made on horses; but, as our readers will perceive, we have observed, in our review of that work, the force of the current of blood in the carotid of a horse must have been sufficient to preserve the permeability of the vessel, when only resisted by the effusion of lymph from inflammation. The experiment of Mr. Hunter, referred to by Mr. Crampton, to which we have directed the reader, shews that bare exposure of the tibial artery, without any ligature, and, consequently, without any effusion of lymph from the divided surfaces of the artery of a dog, was sufficient to induce contraction, and, probably, had the experiment been continued, an obliteration of the cavity. It is true, Mr. C. imputes this to the "degree of inflammation excited, and to the thickening of the coats;" but Mr. Hunter speaks of *contraction*, and, though he afterwards opened the vessel, we read nothing about thickening or inflammation.

Our reason for so particularly calling the attention of the reader to these points is, that, as we remarked of Dr. Jones, so we now repeat it of Mr. Crampton, we conceive both have allowed too little to the resources of Nature. If we were to give a history of the obliteration of an artery, under either of the circumstances now mentioned, we should say, say, that, such is the provision made for the condition of a vessel which can no longer be rendered serviceable for its original purposes, and the failure of which, without such a provision, would end in the death of the animal. By Mr. Jones's other experiments, we see the exact powers of the œconomy defined. If an artery is only so cut that it may be restored, then the attempt at restoration was made, and succeeded in the instances produced in that gentleman's experiments. If the section encroaches to a certain degree on the diameter of the vessel, all attempt at restoring it is given up, and ulceration takes place, as Dr. Jones conceives, in order to enable the artery to contract, "after which the same process follows to suppress hæmorrhage as in other divided arteries."

But, by Mr. Hunter's experiment on the dog, it appears, that no retraction is necessary, nor the division of the artery either entirely or in any proportion of its diameter, nor even the division of its internal coats; but that, if an artery is reduced to a certain condition, the stimulus of imperfection, as, perhaps, Mr. Hunter would call it, is sufficient to induce the attempt at its obliteration.

It should be understood that our object in all this is to direct our younger readers, in all their experiments, to follow Mr. Hunter's plan of studying the resources of the œconomy or its actions under every change. For want of this, we are perpetually prescribing laws to Nature. Thus Sir Everard Home conceives, because *he* knows no other way of making fat but by placing animal matter on the *banks of a common sewer*; that there can be no other power in the living body, Mr. Brodie supposes, because an animal grows cold when his head is cut off; therefore that the brain is the source of heat. Though Mr. Crampton has kept free of these more palpable errors, and satisfactorily shewn that pressure on an artery is sufficient to produce its obliteration, yet, as the artery was first denuded, it remains to be ascertained whether such denudation would not be sufficient of itself, as was ascertained in the case of the dog; and, if denudation is insufficient in the human race, it remains to be proved whether pressure, without denudation, may not be contrived in other instances as well as that to which we have referred our readers, in which a spurious aneurism, occasioned by puncturing the brachial artery, was cured by an obliteration of the vessel above the injury, without any other operation than pressure through the sound integuments and against the bone.

We have already apprised our readers of the importance of this article, and the number of questions involved in it.

 To

To most of these we have given our best attention; and one very serious defect we are obliged to mention a second time. To quote an author from memory, is always improper; but, most of all, when the object of our quotation is to support a favourite opinion. We have already mentioned our objection to the construction of a passage from Mr. Hunter, and to the impossibility of the reader's turning to a passage not particularly cited in a quarto volume of between five and six hundred pages. Another authority, not less objectionable, occurs at page 358, where Mr. Crampton, speaking of Dr. Jones's very valuable experiments, adds,

"It is to be observed, that the division of the internal and middle coats is the fundamental principle of this operation; and I have endeavoured to shew, that this division of the internal and middle coats is precisely the *kind* of injury which a diseased artery is least able to bear with impunity: accordingly, secondary hæmorrhage, *or even aneurism,** is not an unfrequent consequence of this operation."

Here it must be admitted we have a reference to a passage; but, in the 2d edit. of Warner's cases, the only one to which we can refer, page 9, relates a case of the operation of trepanning over the lambdoïdal suture. The only case we can meet with which bears exactly on Mr. Crampton's observation is case xii. page 50; and here we shall see Mr. Warner imputed all his difficulties to a previous disease in the artery, and his subsequent success to fixing his ligature above the disease. As the book is not in every hand, and the case short, we shall transcribe it.

"*Case of an extraordinary Disease of the Humeral Artery.*

"When a bone, and its neighbouring tendons and ligaments, are affected with inflammation, caries, &c. the disease may sometimes extend itself farther, so as to affect the neighbouring vessels; or it may probably happen, that the diseases of these particular parts may sometimes proceed from a previous affection of those very neighbouring vessels, from which they receive their nourishment and growth.

"C. D. was afflicted with a caries of the joint of the elbow, which was attended with such circumstances as rendered the amputation of the limb necessary. The operation was performed at a proper distance above the diseased part, and the vessels were taken up by the needle and ligatures.

"In a few days after the operation, the humeral artery became so dilated above the ligature, as to endanger its bursting. Upon this account, it was judged necessary to perform the operation for the aneurism, which was done, and the vessel secured by

* "See p. 9, Warner's case, &c."

 ligature ligature above the upper extremity of its distended coats. After this, every thing went on seemingly well for some time, when suddenly the artery appeared again dilated, and in danger of bursting above the second ligature. These circumstances made it necessary to repeat the operation for the aneurism: from this time every thing went on successfully, till the stump was at the point of being healed; when, quite unexpectedly, the artery appeared a third time diseased in the same manner as before; for which reason a third operation for the aneurism was determined upon, and performed.

"The last operation was near to the axilla, and the patient continued well from this time without any relapse.

"*Query.* Could the several aneurisms of the humeral artery be attributed to the sudden check alone which the blood met with from its extremity being secured by ligature; or is it not more reasonable to suppose, that the coats of the artery, nearly as high up as the axilla, were originally diseased and weakened? The latter seems the most reasonable way of accounting for these successive returns of the disease of the vessel; since it has been found from experience, that such accidents have been very rarely known to occur after amputations, either of the arm or thigh, where nearly the same resistance must be made to the circulation in every subject of an equal age and vigour, who has undergone the same operation."

We shall forbear any comments on the inference drawn by Mr. C. from such a case, or from a book containing such a case. However, we must not dismiss old Warner, as some will call him, nor his cases, without referring to one object expressed in the title-page, viz. "An Account of the preparation and effects of Agaric of the Oak in stopping Bleedings after some of the most capital Operations." So well satisfied is the author with this mode of "stopping bleedings," from the success of several cases, that he at last attempted it in the femoral artery. Here he failed; but, as he succeeded in every amputation below the knee and above the elbow, and cites the authority of a French surgeon for his success above the knee, the postscript to this edition concludes with the author's determination to make a second attempt.

We pretend not to ascertain the virtues of agaric, but we sincerely wish the powers and resources of the œconomy were more attended to, and that, along with the brilliancy of operations, we heard more of the means by which they may be rendered less necessary.

*Case of Inguinal Aneurism, cured by tying the external Iliac Artery.* By John Smith Soden, Esq. Surgeon to several Institutions at Bath.

All we shall remark of this case is, that the ligature was was *very thin*, and *of silk*; consequently, we must suppose, as the pulsation in the tumour below immediately ceased, that the pressure was very tight, and sufficient to divide the internal coat of the artery. The ligature was detached on the fifteenth day; the upper part of the wound healed in a month, leaving below a small part of the size of a sixpence, which was not completely cicatrized till the seventh week.

*Further Observations on Contractions succeeding to Ulceration of the Skin.* By HENRY EARLE, Esq. Assistant-Surgeon to St. Bartholomew's Hospital.

These additional cases do credit to the industry and ingenuity of Mr. Earle. All here related are the effect of burns, the granulations from which, and even the cicatrizations, are known to contract—the first with a rapidity equal to their formation; the latter for a long time after the new skin is completed. Mr. Earle, by pursuing the plan he before proposed, has restored three distorted hands to usefulness and comfort. A case is added from Mr. Ring, and another from Mr. Hodgson.

*Case of Hernia of the Dura Mater connected with Hydrocephalus Internus.* By HENRY EARLE, Esq. &c.

This hernia consisted of an incysted tumour, which was punctured about a week after birth. As the tumour filled, it was evacuated several times. The child died. A record of such a case is important, inasmuch as it authorizes every practitioner at a distance from the metropolis to undertake a similar operation, and is a sufficient apology should it prove unsuccessful.

*Description of an Extra-Uterine Fœtus contained in the Fallopian Tube.* By GEORGE LANGSTAFF, Esq. Surgeon.

Such cases are more important to physiology than practice; for, as Dr. Clarke and the author observe, "Even had the case been known, we could only deplore the insufficiency of our art to remedy a situation so uncommon and so fatal." We shall, therefore, only notice those particulars in which Mr. Langstaff's case differs from others of fœtus lodged in the fallopian tube; and, as we can neither improve nor shorten the author's description, we shall give it in his own words.

The woman died seven hours after an attack of violent pain. On opening the abdomen, nearly two quarts of apparently arterial blood were taken out. Aneurism was suspected particularly, as Mr. L. had recently discovered such a cause of death in a subject in whom it was never suspected during during life. Finding the fallopian tube dilated to the size of a hen's egg,

"I brought (says Mr. L.) the uterus home, that I might deliberately search for the cause of the increased fallopian tube, and notice such other appearances as might present themselves.

"The figure of the tumour, the appearance of coagulated blood beneath its serous membrane, and its having been lacerated, made it look very like a ruptured aneurism.

"The spermatic artery on this side, and those arteries ramifying between the laminæ of the peritoneum, which form the ligamentum latum, and supplying the fallopian tube, being greatly enlarged, induced me to place pipes in them, and inject some size and vermilion, previous to opening the tumour. The injection was observed to flow in a full stream from the lacerated openings in the fallopian tube, when it was thrown into those vessels; and there was also a moderate stream when the spermatic artery was injected, which proved that the giving way of those arteries was the immediate cause of the person's death, and that the spermatic arteries, besides supplying the ovaria, anastomose freely with the uterine vessels, originating from the internal iliacs.

"The distension of the tube had commenced about two inches from the fimbriated extremity, this part of the canal being rather more capacious than that of the left side; but the internal surface retained its plicated arrangement, whilst the remaining part of the tube, from the enlarged portion to the angle of the uterus, was completely obliterated.

"The lacerations were in the posterior surface of the tubal enlargement, and in the longitudinal direction, each measuring about a quarter of an inch. The fallopian contents were next minutely examined; and, after carefully washing away the coagulated blood from beneath the peritoneal covering, I discovered a chorion and amnios, with a fœtus of about eight weeks, floating in the liquor amnii.

"In consequence of the dilatation of the fallopian tube by the increasing size of the ovum, its different tissues (except the external containing one, which was rendered extremely thin) had been nearly destroyed.

"The right ovarium was made very red with the injection, more especially about the corpus luteum, which was remarkably large, and contained a gelatinous looking mass.

"There were several corpora lutea in the left ovarium, one of which shewed the vesicle having been removed from thence in the conception previous to the last.

"Lastly, the uterus was examined; it was considerably larger than we generally observe that organ to be in the unimpregnated state, even in women who have borne several children. On laying it open, the uterine vessels were observed to be very large, but empty; and there was a great quantity of gelatinous matter in the cavity and neck of the uterus. When this was washed off, the internal internal surface of the viscus looked very vascular, having been highly injected; but there was not the least appearance of a decidua.

"The cervix uteri was not closed by the mucous secretion from the glands situated there, as it is during utero-gestation, when it proceeds in the ordinary manner; as I easily introduced my finger through it into the uterus, although the lacunæ were numerous and loaded with mucus."

Some valuable observations follow, particularly on the condition of the uterus, its mouth remaining open; and its size, which, though enlarged, was not equal to one containing a fœtus of that age; and, lastly, the absence of a membrana decidua.

"Perhaps (says Mr. L.) the gelatinous matter found in the uterus may, by some gentlemen, be considered as the incipient step towards the formation of that membrane.

"If it be admitted that this membrane is an adventitious production of the internal surface of the uterus; that in the mode of production it resembles the laminæ of coagulating lymph, formed by inflamed surfaces; and that it is produced previous to the reception of the ovum into the uterus, I think I shall not be considered incorrect in having stated, in the history of the dissection, that a decidua was not present.

"In the cases of ruptured fallopian tube from extra-uterine conception, which have been related, mention is only made of the laceration, without noticing the morbid changes produced in that part, and the probable cause of its sudden rupture.

"Dr. Clarke merely says, 'But the most remarkable thing in this case is, the laceration of the fallopian tube, and the fatal hæmorrhage thereby occasioned.' Dr. Burns thinks the tube slowly inflames, and sloughing takes place. Having only seen this solitary case, I shall not presume to say what takes place generally in those instances; but shall venture to describe what appears to me, from the result of the dissection, the most probable cause of laceration.

"In consequence of the regular developement of the ovum, the containing parts are distended beyond their capability, and become attenuated; progressive absorption takes place; the vessels supplying the ovum, which are necessarily enlarged, suffer in the destructive action; and, from their not having been obliterated by adhesive inflammation, their coats are gradually absorbed, they burst, and the blood now finding free access to the sac, which is already rendered extremely thin, it bursts as in aneurism, the blood is propelled into the cavity of the abdomen, and thus life is extinguished.

"One of the most remarkable, and, I believe, unique, circumstances in this case is, that no communication existed between the uterus and the ovum, the canal of the tube being completely obliterated; and it is also worthy of remark, that there was only the corpus corpus luteum in the right ovarium, which has been described,—all the ova except this having appeared to be produced by the opposite ovary.

"It would be impossible to state accurately, whether this obstruction took place before the fallopian impregnation, or in consequence of it; but, in a physiological point of view, it would be very desirable to ascertain this point, as it would satisfactorily account for the detention of the ovum, and refute the opinion entertained by some physiologists, that the semen must be in actual contact with the ovum for the production of the species.

"From the complete state of obliteration of the tube, I must confess I feel inclined to suppose, that it was not effected by adhesion in consequence of inflammation from the last conception; or else, from the same cause, why was not the extremity of the tube obliterated?"

From this specimen of Mr. Langstaff's observation and reasoning, we wait with much anxiety for his promised case of aneurism, announced in the paper before us.

*On the Treatment of Sinuous Ulcers.* By HENRY DEWAR, M.D. F.R.S. Ed. &c.

Nothing can be more pleasing than the restoration of some of those chirurgical therapeutics, which, among the moderns, are, in too many instances, superseded by the brilliancy attending operations. We mean not by this to undervalue Dr. Dewar's *discovery*, for such we consider it, if (as we have no reason to doubt) his proposed plan has not been fairly attended to between the time of Galen and Heister, and altogether neglected since the writings of the latter. We say we consider it a discovery, not because it is not to be met with in systems of surgery, cyclopædias, complete practices, and *vade-mecums*—most of these books, by the slovenly manner in which they are composed, usually by writers without experience, and, consequently, unincumbered with doubts or difficulties, have proved the bane of modern practice in all its branches. But Mr. Dewar informs us that his plan is neither taught in the schools, practised in public hospitals, nor described in any recent publications. Whilst we give the author credit for so much merit, we are forced to acknowledge that his paper might have occupied fewer pages. It is, however, well written; and who shall accuse the discoverer of any improvement, if he shows some anxiety to be well understood, when he tells us,

"Both the patient and the medical gentleman who saw it were surprised at the celerity of the cure. But it was not, to my knowledge, made the subject of any discussion or conversation, farther than by being mentioned as a *remarkably fortunate case.* The only circumstances, then, systematically attended to in the application of bandages, were the neatness and smoothness of their appearance, and the utility of the uniform pressure recommended by Mr. Baynton in superficial ulcers. A pressure greatest at the remote part, and gradually declining towards the outlet of a sinus, to give the compressed fluid a safe direction, and to secure the commencement of adhesion in that quarter in which it is not liable to future interruption, though obvious dictates of surgical science were never thought of."

Having given this apology, we shall proceed, in the author's words, to a description of his mode of applying pressure. After objecting, with much propriety, to the common mode of bandaging a limb containing sinuous ulcers, he adds,

"The following is the method to be substituted for the preceding expedients. A few turns of the roller should first be made *with considerable pressure* over one extremity of the femur, and then over the other, so as to reach with all possible certainty the extremities of the large sinus, into which the whole cellular interstices of the parts have been converted. It is safer to begin beyond the sinus than to run any risk of falling short of its extremities; and, in some cases, it might be proper to increase our security by means of partial compresses extending somewhat higher than it is possible to apply the turns of the roller itself. It is now fixed in its situation with a pin. A considerable pressure is easily borne, as no high inflammation is present; and the evacuation of the pus, by reducing the circumference of the limb, soon relieves the veins from any turgescence arising from the pressure to which they may have been at first subjected. In country practice, when a surgeon has been newly called to an old case of this kind, and a considerable interval may elapse before he is to repeat his visit, the swelling of the lower part of the limb may be obviated by bandaging it upwards from the toes. After fixing the bandage on the thigh at the degree of pressure which I have described, the surgeon may, if he chooses, make two or three lighter turns on the tumid part to assist the depletion of it; taking care that these press so lightly, as in no degree to counteract the operation of the first turns made at the extremities of the sinus. The change which this application produces is almost immediate. Part of the matter with which the integuments had been distended is irresistibly forced a certain way towards the orifice; and no newly-secreted matter is suffered to lodge in that quarter. On the second day, the limb is found somewhat reduced in size, and the bandage may now be applied more extensively. On the third day, it may be so applied as to be kept on for several days without alteration. The same degree of pressure is always to be continued over the extremities of the sinus, and several additional turns are to be made, gradually looser, alternately above and below the orifice, and approaching to it in both directions, but not reaching it. If there are two orifices, one of them, by which the matter can be freely brought brought away, is to be left uncovered with the bandage, and the other allowed to heal up. There is no necessity for selecting the most dependant one for that purpose, as any advantage derived from the tendency given to the course of the matter by its own weight, is not worthy of attention under a treatment implying means of evacuation otherwise so powerful. The anterior orifice will often be found the most eligible, as it is examined and dressed with greatest convenience. During the alternate application of the bandage to the higher and the lower part of the thigh, it is frequently and variously *crossed* on the side of the limb opposite to the open orifice, and thus a propulsion of the pus is commanded in every direction to that outlet. A considerable part of the surface surrounding it is left uncovered, and the bandage is finally fixed. Over the orifice such light dressings are subsequently applied as will make no resistance to the discharge of the purulent matter. The firm propelling bandage is kept on without alteration, except when it becomes loose in consequence of a reduction in the size of the limb; although cleanliness requires the dressing immediately over the orifice to be changed daily or oftener. Thus all unnecessary trouble is prevented, an object which is sometimes of importance in securing the more perfect performance of those offices which are really necessary.

"When no further cause of disease has existed besides those now mentioned, and when the pus is the only foreign body contained in the cavity, the process of adhesion soon begins at the extremities of the sinus. The principles of adhesion have been very well understood since Mr. John Hunter wrote on the subject; but it will be useful on the present occasion to remark, that a considerable pressure over relaxed parts promotes it independently of the evacuation of pus, by affording mechanical support, in the same manner as it promotes the healing of the relaxed surfaces of ulcers. It thus supersedes the stimulant injections which have been so often employed for the purpose of inducing rawness. The process of adhesion advances by successive steps from the extremities towards the orifice of the sinus, which, even in the end, requires but little pressure. The discharge quickly diminishes, and shews a proportional diminution of internal disease; and the parts heal in the same kindly manner as a superficial abscess among firm integuments after the pus has been discharged. In the case to which I have alluded, these effects were amply and speedily obtained."

Some learned references follow from Galen, Aëtius, Paul. Ægineta, Heister, Tagault, and Fabricius; after which Mr. D. proceeds—

"The mode of bandaging now described will possess considerable advantages in cases of more complicated disease. If pieces of cloth, splinters of bone, or other foreign bodies, are present, it will tend to expel them by the ready evacuation which it effects. If it does not expel them, yet, by reducing the local disease to the spot in which they are lodged, it procures for the practitioner sure

 information information of their presence, and thus gives him an opportunity of using his best endeavours to remove them. In all cases, it must facilitate recovery by circumscribing the local disease: even where the bone is fractured by a musket shot, it will have the effect of reducing the disease more nearly to that of a common compound, and soon to that of a simple fracture.

"When the discharge from a sinuous ulcer is fetid and ichorous, shewing an unhealthy state of the parts, some might be disposed to expect less advantage from this mode of bandaging. This most frequently occurs among numerous tendons or ligaments, as in the ham or in the foot; and arises, in some measure, from the nature of that species of structure, and the forcible separation of the parts attending it: and the disease will, in some degree, yield to the powerful compression of a bandage diminishing the contrast between the firmness of the ligaments and the openness of the intervening spaces. In many cases, a healthy action may thus be established, especially if the aid of stimulant injections is superadded.

"It is scarcely necessary to describe the modes in which the same principles may be applied to the treatment of sinuses in other parts of the body. In the arm and leg there will be no occasion to deviate from the exact form of the bandage as already described. When a sinus exists under the integuments of the foot, extending among the ligaments and tendons, many respectable surgeons lay them open the whole length of the foot, and then use means for healing up the cavity by granulation. This harsh operation may evidently be superseded by the bandage applied according to the principles above explained, and with a tightness proportioned to the resistance which it receives from the structure of the parts.

"In sinuses occurring among the integuments of the trunk, greater address is required, and, in many cases, the success of the most judicious management must be uncertain, as we have not such points of support as will afford complete security against the extension of the sinus to the great cavities of the body. In some cases, a compress in the form of a crescent might prove useful for preventing the spreading of the suppuration in various directions, and thus promoting the tendency of it to the orifice. The open part between the horns of the crescent will correspond to the outlet."

Such is the general purport of this ingenious paper, by which, we doubt not, this teazing part of surgery will be much simplified. Respecting the pathological reasoning, may we venture to express a doubt whether the author has given sufficient credit to some degree of inflammation which his mode of pressure may induce, and which may further assist the process of adhesion? We are far from lessening the merits of this discovery by this or any other remark.

Such are the contents of the second part of this volume. Our remarks sufficiently show how much we conceive it to exceed the first, particularly in the chirurgical department.

*An*

*An Essay on the Mode by which Constitutional Disease is produced from the Inoculation of Morbid Poisons.* By CHARLES SALT, Member of the Royal College of Surgeons, London. 8vo. pp. 88. Cox and Co.

THE author's intention in this tract is, "if possible, to ascertain why these poisons *appear* to be exempt from the immediate agency of the absorbent system," considering "that the mode of explanation by fermentation, nervous sympathy, or a supposition that these poisons remain a long time inert in the inoculated part, rather appears to increase the difficulty than to afford any satisfactory explanation."

The author's theory follows:—

"When a morbid poison is applied to an abraded surface, it has a specific power inherent in it of exciting a new secretory action in the *very contiguous* arteries, by which a poisonous fluid is secreted, which, if absorbed from the part, is capable of infecting the individual in whom it is produced, *directly* by such absorption; and of infecting any other individual *indirectly*, viz. by a repetition of this process in the part of his body to which it is applied. This process is never immediate, but slow; and is preceded and accompanied by local inflammation."

After a few other remarks, to shew that the process is not immediate, and that the matter is not absorbed till after a new local action is excited in the part to which it is applied, the chapter concludes with the following summary:

"1st. After inoculation, simple absorption of the deposited fluid is found incapable of producing constitutional disease, without the intervention of a local secretory process.

"2d. That constitutional effects (as far as experience allows of a decision) may be always prevented by excision or destruction of the contiguous organized structure, antecedently to the local suppuratory process.

"3d. That, from general experience, where the primary suppuration or secretion goes through its regular process, the constitutional disease, amongst individuals capable of receiving the complaint, may be always expected to follow."

A chapter follows on the functions of arteries and absorbents, in which the arteries are considered as the suppuratory organs. We were in the habit of imputing it to glands. "A bullet," says Mr. Salt, "may remain in a part, in some instances, for years, and then produce a secretion of pus, which, by making its way to the surface, gives an opportunity for the escape of the ball." Now, we are rather led to suppose that this process of suppuration does not commence till the ball arrives near the surface. Such is the case with other substances, which, having been swallowed, lowed, pass through a considerable part of the body without inducing suppuration in parts in which suppuration would, probably, be fatal; but, as soon as they approach the surface, suppuration commences, an abscess is formed, and, without any previous suspicion, a pin, or some other sharp body, appears.

Some general remarks follow to show that "the arteries, once having received their specific impulse by the inoculation of morbid poisons, after some time, secrete a fluid in each case, *sui generis*, which is capable of producing the same disease, under certain circumstances, in others."

An objection occurs in hydrophobia, beyond comparison the most important of all; but, in other morbid poisons, there is this difference between the matter reproduced by local secretion, before its removal from the body of the patient, and subsequent to such removal. In the former case it has the property of infecting the person in whom it is secreted by direct absorption, and in the latter it possesses no such power. That is, if we understand the passage, the local action must be excited before the constitution can be affected. This is illustrated by what the author considers a sufficient proof.

"It may, perhaps, be said, how absurd to suppose that a secretion, while enclosed in the part in which it was secreted, should possess a property which it was unable to retain after its removal!

"But, let it be remembered that there is a large class of plants which blossom only once before they perish; and the plant, antecedently and subsequently to flowering, may be said to possess different powers or faculties. No man would plant annuals in his garden which had blossomed.

"Perhaps a closer analogy may be obtained from the history of some animal fluids. The blood, for example, while circulating in the living animal, possesses certain known properties essential to the continuance of life; remove it from the body, and, in a very short time (perhaps in a few seconds), it loses the peculiar powers and properties it possessed when in a state of circulation. If such blood be returned into the veins of an animal, it becomes, from the change it has undergone, a mere extraneous matter, no longer capable of the performance of its usual functions."

We cannot easily see the necessity for these analogies: the fact must rest on observation, and is, we believe, pretty generally admitted. But we know not on what authority the glanders and farcy in horses are said to be excited by the same matter, differently applied. We are referred, it is true, to Mr. White's experiments, from which extracts are given; but these extracts are not satisfactory, till it is found that the matter of farcy will excite glanders.

The rest of the work goes to show that, if, after inoculation of any morbid poison, the part is cut out, no ill consequences sequences will follow, but such as arise from a clean cut. In hydrophobia some difficulties occur.

We are much pleased with the good intentions of Mr. Salt, and hope he will pursue all the experiments he speaks of; after which we shall be very thankful to learn the result. One caution, however, we are obliged to repeat, viz. that, though the object of his experiments may be to shew that all morbid poisons, inducing local action, are governed by the same laws, yet his business must be to detect all the actions induced by them, before he confines them to laws. When he has discovered the actions of each by the forms they exhibit, he is then in possession of the laws of each, and may easily discover how far they are general to all, or similar in each; and, if not so, he may inform us in what respects they agree, and wherein each or either differs.

---

### *Essai sur l'Anatomie Pathologique en general, &c.*

*Essay on Pathological Anatomy in general, and on Transformations and Organic Productions in particular.* By J. Cruveilhier, M.D. 2 vols. 8vo. Paris, 1816.

Pathological Anatomy, or the anatomy of the body in a morbid or diseased state, is a study which has but very recently been cultivated with ardour and success. For example, the first attempt at a system of pathological anatomy dates no farther back than the close of the seventeenth century; since which period many illustrious names, impressed with its importance, have devoted all their attention to it. Hitherto, the authors who have treated the subject have followed in their classifications the order of the regions of the body. This arrangement is defective in many respects: it involves frequent repetitions, and never gives a general or perfect idea of the disorder. M. Cruveilhier follows a different plan (similar to that of M. Dupuytren in his lectures): he separates those parts which have no relation, and unites those which have.

His work is divided into four sections:—The first, *mechanical lesions,* or accidents. The second, *organic diseases.* The third, *organic lesions,* which may be occasioned by— 1st, irritation; 2d, atony; 3d, the *death* of some part of the body. The fourth section contains *vital disorders,* or those in which we have not been able to discover any derangement of the mechanism of nature, and for which pathological anatomy offers no aid, as fevers, &c.

Mechanical lesions comprehend ten classes—1, wounds; 2, ulcers; 3, fistulas; 4, contusions; 5, distensions, ruptures, &c.;

&c.; 6, fractures; 7, the displacing of hard or soft parts; 8, foreign bodies; 9, aneurisms; 10, vices of conformation.

The second section treats of transformations, productions, and degenerate organizations; perhaps, the most important part of pathological anatomy, and the least known. The greater portion of Mr. C.'s work is accordingly consecrated to it. The degenerations form three orders—1, scrofulous; 2, cancerous; 3, from chronic inflammation. The first two orders present many important varieties.

The third section comprises—1, nutritive irritation; 2, secretory, &c.; 3, hæmorrhagial; 4, inflammatory. The atonies are distinguished into—1, nutritive; 2, secretory; and 3, hæmorrhagial. Next gangrenes—1, organic lesion; 2, lesion from circulation or nervous action.

Such is the arrangement of Mr. C.'s work, which, if executed with the talent and perspicuity we might expect from such a writer, merits to be continually consulted as the anatomist's vade-mecum, containing the state of science down to the present hour. Mr. C. is a young author: we believe this is his first work. But he has devoted himself to these studies, with all the advantages he may derive from the too-compressed labours of our Baillie. We are also assured, that his views are frequently original, and that he possesses the happy art of transferring the clearness of his own conceptions to the mind of his reader.

---

*Avis aux Femmes qui entrent dans l'Age Critique, par* CH. P. L. DE GARDANNE, &c. &c.

*Advice to Women at the Critical Period of Life, &c.* By DR. GARDANNE. 1 vol. 8vo. Paris, 1816.

DR. GARDANNE, though very young, has already acquired a high degree of reputation; and his work possesses at least one exclusive merit, that of being the first wholly devoted to a most important and highly-interesting subject. But it were much to be wished that the fire of youth had been a little tempered by the sobriety of age; and Dr. Gardanne would have deserved still better of society, if, instead of addressing his work to the ladies, whom it concerns, he had treated the subject more scientifically, which he is well capable of doing. Works of science should never be written in the language of romance—they lose thereby even the portion of weight they merit. And even the ladies, at that time of life, would, we apprehend, have been better pleased with Dr. Gardanne had he treated the subject with grave importance, than in striving to excel as an agreeable writer.

The

The base of the work is, however, excellent: we have only to find fault with the extraneous and foreign ornaments with which he has thought fit to embellish it. He divides his work into three parts. The first contains an anatomical and physiological dissertation on the uterus at the turn of life.

In the second, the author points out the modifications which the whole constitution of the female undergoes at this period, from which he deduces precepts proper to be attended to in order to prevent the disorders to which women are then subject.

The third part contains dissertations on the disorders incident to that period, with particular observations on the mode of treatment of each. As the work is likely to become popular, not only from its scientific interest, but also as a valuable tribute to the sex, we may expect a second edition with those alterations, which enlightened criticism and the matured judgment of the author shall have dictated.

---

## Figures and Tables

**Figure f1:**